# Validation of a High-throughput, Multiplex, Real-time Qualitative Polymerase Chain Reaction Assay for the Detection of Sabin Oral Polio Vaccine in Environmental Samples

**DOI:** 10.1093/cid/ciy639

**Published:** 2018-10-30

**Authors:** Jonathan Altamirano, Sean Leary, Christopher van Hoorebeke, Clea Sarnquist, Rasika Behl, Lourdes García-García, Leticia Ferreyra-Reyes, ChunHong Huang, Marvin Sommer, Yvonne Maldonado

**Affiliations:** 1 Stanford University School of Medicine, California; 2 Instituto Nacional de Salud Pública, Cuernavaca, Morelos, Mexico

**Keywords:** OPV, poliovirus, environmental surveillance, RT-qPCR, Mexico

## Abstract

**Background:**

Currently, the primary mechanism for poliovirus detection is acute flaccid paralysis (AFP) surveillance, with environmental sampling serving as a complement. However, as AFP cases drop, environmental surveillance will become increasingly critical for poliovirus detection. Mexico provides a natural environment to study oral polio vaccine (OPV) transmission, as it provides routine injected polio vaccine immunization and biannual OPV campaigns in February and May.

**Methods:**

As part of a study of OPV transmission in which 155 children were vaccinated with OPV, monthly sewage samples were collected from rivers leading from 3 indigenous Mexican villages (Capoluca, Campo Grande, and Tuxpanguillo) from February to May 2015. Samples were also collected from October 2015 to October 2017, during which time there were standard OPV campaigns. Samples were analyzed for the presence of OPV serotypes, using a real-time qualitative polymerase chain reaction assay capable of detecting as few as 9, 12, and 10 copies/100 µL of viral ribonucleic acid for OPV serotypes 1, 2, and 3 (OPV-1, -2, and -3), respectively. Included here are 54 samples, taken up to November 2016.

**Results:**

Of the 54 samples, 13 (24%) were positive for OPV. After the vaccination of 155 children in February 2015, OPV was found 2 months after vaccination. After unrestricted OPV administration in February 2016, OPV was detected in sewage up to 8 months after vaccination. OPV-3 was found in 11 of the 13 positive samples (85%), OPV-2 was found in 3 positive samples (23%), and OPV-1 was found in 1 sample (8%).

**Conclusions:**

OPV can be detected even when small amounts of the vaccine are introduced into a community, as shown by OPV-positive sewage samples even when only 155 children were vaccinated. When OPV vaccination was unrestricted, sewage samples were positive up to 8 months after vaccination, implying community OPV circulation for at least 8 months. OPV-3 was the serotype most found in these samples, indicating prolonged transmission of OPV-3 when compared to the other serotypes. Future work could compare the phylogenetic variance of OPV isolates from sewage after OPV vaccinations.

Since the inception of the Global Polio Eradication Initiative in 1988, paralysis due to wild poliovirus (WPV) has declined by more than 99%, from roughly 350000 cases to only 22 cases in 2017 [1, 2]. With the last recorded case of WPV serotype 2 isolated in 1999, WPV serotype 2 was declared eradicated in 2015: the first pathogen eradicated since smallpox [1]. Additionally, WPV serotype 3 has not been detected since November 2012, leaving only WPV serotype 1 endemic in only 2 countries, Afghanistan and Pakistan [3]. Thus, WPV eradication may be achieved in the next few years.

There are 2 polio vaccines currently in global use: (1) live, attenuated oral polio vaccine (OPV) and (2) inactivated, injected polio vaccine (IPV). OPV has been the primary vaccine used to immunize children against WPV, especially in low- and middle-income countries, due to its low-cost, easy administration and its ability to provide community immunization via fecal-oral transmission to household and community contacts of vaccinees [4]. However, OPV itself can cause vaccine-associated paralytic polio, estimated to occur in 2–4 cases/1000000 live births per year in countries that use OPV [5]. Of further concern, long-term replication of OPV and mutation of the virus can result in genetically divergent vaccine-derived poliovirus (VDPVs) [6]. Circulating VDPVs (cVDPVs), or VDPVs that show evidence of prolonged community circulation, are shown to cause paralysis that is indistinguishable from WPV [6]. In 2017, there were 96 cases of cVDPVs, almost 20 times the number of cVDPV cases in all of 2016 [2].

Currently, polio endgame strategy uses acute flaccid paralysis (AFP) surveillance to detect poliovirus, with environmental surveillance serving as a complement for detection [7]. However, most poliovirus infections are asymptomatic, with only 5% of infections resulting in clinical symptoms and only 1% resulting in paralysis. In contrast, environmental surveillance, while resource intensive, has successfully been used to identify cases of WPV and VDPVs in sewage and other environmental samples, even in the absence of AFP cases [8]. For example, 3 months before AFP surveillance successfully identified a serotype 1 outbreak, India’s environmental surveillance program was able to detect WPV serotype 1 [9, 10]. Similarly, environmental surveillance was used in Nigeria to identify prolonged wild-type serotype 1 circulation and the presence of cVDPV serotype 2, which were missed by routine AFP surveillance [8].

Previous studies have shown that OPV can circulate in the environment for close to a year. New Zealand switched from OPV to IPV in February 2002 and tested sewage for 13 months post-transition. Sewage OPV positives were found monthly through May 2002, and then sporadically until January 2003 [11]. Indonesia started an environmental surveillance program in 2004 and switched from OPV to IPV in September 2007. Sewage tested positive for OPV for just 6 weeks after the switch [12]. Sewage samples were studied in Mexico following the Mexico May 2010 National Immunization Week (NIW). Collection continued after the February 2011 and May 2011 NIWs, and OPV positives were found every month until collection ceased in July 2011 [13]. A similar study was performed in Cuba after a 2003 national immunization campaign and found OPV positives in sewage for 15 weeks following the campaign [14]. As the number of AFP cases continue to decline, environmental surveillance will become an increasingly critical tool for poliovirus detection.

In order to improve poliovirus surveillance methodologies, we describe here the validation of a highly-sensitive real-time qualitative polymerase chain reaction (RT-qPCR) assay for use in the detection of OPV in environmental samples collected monthly from 3 rural, indigenous Mexican communities. Mexico serves as a natural environment to study OPV, due to its unique vaccination policy, in which children are both routinely vaccinated with IPV and also with OPV during bi-annual NIWs in February and May.

## METHODS

### Environmental Sample Collection

As part of a larger study of household and community OPV transmission [15, 16], sewage samples were collected monthly from 3 geographically-isolated, rural, indigenous Nahuatl villages in the Ixtaczoquitlán municipality of Orizaba, Veracruz, Mexico. These samples were collected before, during, and after the OPV vaccination of 155 children across all 3 villages. The details of this study have been published previously [16]. Sewage flows away from each village via streams that travel from the villages and into a major river. One sewage collection site was identified per village, and in each case was located where all streams combined before reaching the major river. Initially, 4 sewage samples were collected from each village, 1 sample per month, from February–May 2015 as part of the larger study on OPV circulation. After May 2015, sewage collection was halted until October 2015, after which a sample was collected from each village every month until October 2017. This sample collection included the last time trivalent OPV (tOPV) was used in Mexico, in February 2016, as well as the first time bivalent OPV (bOPV) was used in Mexico, in February 2017. A total of 87 samples were collected.

Each 1-liter sewage sample was collected in sterilized glass flasks and transported to a laboratory in Orizaba, Mexico, for processing. At this laboratory, the sewage was divided into 100 mL aliquots and concentrated with a vacuum filtration method at 15 psi using Millipore HAWG 0.45 micron filter membranes (Millipore Inc, Billerica, MA). Filters were then transferred to 2 mL cryovials containing 500 μL guanidine isothiocyanate (GITC), which were labeled with unique IDs via barcode. These vials were then stored in cryoboxes with 81 numbered slots at -70°C, until they were shipped in dry ice to the Stanford University laboratory, where they were maintained at -80°C until tested. A total of 870 aliquots—10 aliquots per sewage sample—were processed.

### Environmental Sample Assay Validation Methods

Untreated wastewater was obtained from the Palo Alto Wastewater Treatment Facility using the grab method (a bucket attached to a rope). The wastewater was chilled at 4°C and brought back to the laboratory to be stored at -80C until processing. The wastewater was separated into 50 mL aliquots to be vacuum filtered using a 0.45 uM membrane. The membranes were used to filter 2 aliquots of 50 mL each at the same time (filtering 100 mL of wastewater total).

A synthetic composite oligonucleotide standard containing 10^9^ viral ribonucleic acid (RNA) copies of Sabin 1–3 was used to create a 9-fold 1:10 serial dilution, ranging from 10^8^ copies/reaction to 1 copy/reaction. Stock viruses, identical to those previously described, for each of the 3 Sabin serotypes were diluted in a 12-fold 1:2 serial dilution [17]. In order to determine the lower limits of detection for our assay, 10 uL each of OPV serotypes 1, 2, and 3 (OPV-1, OPV-2, and OPV-3) dilution were spiked into the corresponding 100 mL aliquots.

Following filtration, the membrane was placed in 700 uL of GITC buffer and 1 g of ceramic beads was enclosed inside a 2 mL screw-cap tube. The RNA from the filter was extracted and underwent RT-qPCR, as described below.

### Environmental Sample Assay

The sewage samples were tested for the presence of OPV-1, -2, and -3 using a novel RT-qPCR assay. An additional 200 μL of GITC buffer was added to each sample (for 700 μL total) and then thawed for processing. In brief, viral RNA was extracted from the filtration membrane utilizing the MagNA Lyser (Roche), MagMAX Viral RNA Isolation Kit (Invitrogen), and KingFisher Duo Prime (Fisher Scientific) using the bacteriophage MS2 as an internal control for extraction efficiency. Viral RNA then underwent RT-qPCR in order to detect and quantify any OPV serotypes present in the samples. The probes and primers were adopted and adapted from Kilpatrick et al. [17] and the Centers for Disease Control protocol for polio qPCR. Samples were run in triplicate and a sample was considered positive if two-thirds of reactions had a cycle threshold (Ct) < 36. More detail on the RT-qPCR protocol can be found here [18]. The assay results presented here include 540 aliquots from 54 samples collected from February–May 2015 and then from October 2015–November 2016. Sanger sequencing of each sample was performed where possible, in order to confirm that the isolates were OPV sequences.

## RESULTS

### Environmental Sample Assay Validation Results

Across an average of 5 validation tests, we were able to determine the sensitivity of the assay. The average lower limits of detection were determined to be 12, 13, and 13 copies/100 µL of viral RNA for OPV-1, -2, and -3, respectively, and were capable of detecting as few as 9, 12, and 10 copies/100 µL of viral RNA for OPV-1, -2, and -3, respectively (Tables 1 and 2). The lowest detectable copy numbers were determined from the single triplicate across all 5 runs with the lowest copy number of each Sabin serotype.

**Table 1.** Average Copy Number for Each Sabin Serotype Detected

		Average Copy Number	
Run	OPV-1	OPV-2	OPV-3
1	9	12	10
2	13.5	13.5	20
3	14.5	16	12.5
4	12	18.5	13
5	10.5	4.5	8
Mean	**12**	**13**	**13**

Shows the average LLOD for 5 separate runs for each OPV serotype. The average of the 5 runs shows the LLODs to be 12, 13, and 13 copies/100 µL of viral RNA for OPV-1, -2, and -3, respectively, as highlighted in bold.

Abbreviations: LLOD, lower limits of detection; OPV, oral polio vaccine; RNA, ribonucleic acid.

**Table 2.** Lowest Validation Run Results

	OPV-1		OPV-2		OPV-3	
Dilution	~copy #	Ct	~copy #	Ct	~copy #	Ct
1	4608	27.82	6144	28.01	10240	27.02
2	2304	28.80	3072	28.53	5120	27.81
3	1152	29.53	1536	29.26	2560	28.48
4	576	30.37	768	30.52	1280	29.31
5	288	30.96	384	30.97	640	30.03
6	144	32.61	192	32.26	320	31.31
7	72	33.08	96	32.74	160	31.6
8	36	34.27	48	34.23	80	32.17
9	18	35.75	24	34.78	40	33.25
10	**9**	**36.40**	**12**	**36.07**	20	35.34
11	5	0.00	6	37.77	**10**	**36.89**
12	2	38.33	3	37.00	5	37.11

Positive results were defined as a Ct < 37 in the validation. The copy number and Ct averages of the triplicates are indicated above for each OPV serotype. For this particular run, the lower limits of detection were 9, 12, and 10 copies/100 µL of viral RNA for OPV-1, -2, and -3, respectively, as highlighted in bold.

Abbreviations: Ct, cycle threshold; OPV, oral polio vaccine; RNA, ribonucleic acid.

### Oral Polio Vaccine Detection in Environmental Samples

Of the 540 aliquots analyzed, 15 (2.7%) were positive for OPV. These 15 aliquots came from 13 different samples, with 2 samples having more than 1 positive aliquot. During the initial collection period, February–May 2015, we found 2 positive samples: (1) in February 2015 before OPV vaccination and (2) in April 2015, 1 month after OPV vaccination. After collection resumed in October 2015, OPV wasn’t detected again until February 2016, 1 month before the February 2016 NIW. After OPV administration, OPV was detected in sewage 1–6 months after vaccination, as well as 8 months after vaccination (Table 3). Eleven of the 15 positive aliquots were positive for OPV-3. OPV-2 was found in 3 of the 15 positive aliquots, while OPV-1 was the least detected, and was found in only 2 aliquots from the same positive sample (Table 3). Of the 15 positive aliquots, 5 (33%) were successfully Sanger sequenced, and all 5 detected OPV sequences.

**Table 3.** Sewage Sample Analysis Results

	Study Communities OPV Isolate Detection		
Sewage Collection Dates	Capoluca	Campo Grande	Tuxpanguillo
**9 Feb 2015**	Neg	OPV-3 Pos	Neg
OPV Vaccination: February 27–March 10 2015			
**9 Mar 2015**	Neg	Neg	Neg
**9 Apr 2015**	Neg	OPV-2 & -3 Pos^b^	Neg
**9 May 2015**	Neg	Neg	Neg
OPV Vaccination: 23–29 May 2015 Temporary Halt on Environmental Sample Collection (June, July, August, September)			
**9 Oct 2015**	Neg	Neg	Neg
**9 Nov 2015**	Neg	Neg	Neg
**9 Dec 2015**	Neg	Neg	Neg
**9 Jan 2016**	Neg	Neg	Neg
**9 Feb 2016**	OPV-3 Pos^b^	Neg	Neg
OPV Vaccination: 20 February–19 March 2016^a^			
**9 Mar 2016**	OPV-3 Pos^b^	Neg	Neg
**9 April 2016**	OPV-3 Pos^b^	Neg	Neg
**9 May 2016**	OPV-3 Pos	Neg	OPV-3 Pos
**9 June 2016**	OPV-3 Pos^b^	Neg	OPV-3 Pos
**9 July 2016**	Neg	Neg	OPV-2^b^ & -3 Pos
**9 Aug 2016**	Neg	Neg	OPV-3 Pos^b^
**9 Sept 2016**	Neg	Neg	OPV-2 Pos^b^
**9 Oct 2016**	Neg	Neg	Neg
**9 Nov 2016**	OPV-1 Pos^b^	Neg	Neg

Results of real-time qualitative polymerase chain reaction analysis of environmental samples collected from 3 rural, indigenous Mexican communities, from February 2015–November 2016.

Abbreviations: Neg, negative; OPV, oral polio vaccine; Pos, positive.

^a^Final use of tOPV in Mexico.

^b^Sample was PCR positive, but contained too little virus to be confirmed via Sanger sequencing.

## DISCUSSION

Here we present the validation of a novel RT-qPCR assay for the detection of OPV in environmental samples: we used this assay to analyze 54 sewage samples of 1L each, divided into 540 aliquots of 100mL. Our assay validation found limits of detection as low as 9, 12, and 10 copies/100 µL of viral RNA for OPV-1, -2, and -3, respectively. Even so, only 2 of the 13 positive samples had multiple positive aliquots. This result indicates that the filter paper from these aliquots was not homogenous after division and concentration via vacuum filtration. In future environmental testing, if samples are divided across multiple aliquots, then all aliquots need to be tested for the presence of OPV. This will be of particular importance in future polio surveillance to ensure that low levels of OPV circulation are not missed.

During our prospective study of household and community transmission, only 1 environmental sample was positive after administration of OPV: one month after vaccination in April 2015. While only one sample was positive, only 155 children were vaccinated across all 3 communities. With 1026 children ≤5 years old in these communities at the time of the study [16], we only vaccinated 15% of the children who would otherwise be eligible for OPV. However, with such low coverage, we were still able to detect OPV in our environmental samples. Previous work has shown that as little as 0.01% of a population needs to shed OPV for detection in sewage samples [8]. Our finding here provides further evidence that OPV is detectable in the environment, even when only small amounts of the vaccine are introduced into a community.

We found OPV-positive sewage samples as late as 8 months after the NIW in February and March 2016. This duration is only slightly longer than that found in our previous work in a similar Mexican community, during which OPV circulation ended 6–7 months after the NIW [13]. The duration of circulation detected in environmental samples does vary quite dramatically by location. For example, we found longer detection times than studies in New Zealand and Indonesia that looked at OPV circulation after the national transition from OPV to IPV vaccination, which detected OPV post-transition for up to 12 weeks and 6 weeks, respectively [11, 12]. Differences in OPV detection could have arisen from differences in methodologies, as both the New Zealand and Indonesia studies used cell culture to detect OPV, while we used a new RT-qPCR assay [11, 12]. Differences in OPV detection could have also arisen from the differences in infrastructure between these countries, such as the availability of clean running water and sewage treatments. A final possibility is that the OPV-positive sample from November was imported into the community, as samples from that community were negative from July to October 2016.

OPV-3 was the most-detected serotype and was found in almost every positive sample, while OPV-1 was only found in a single environmental sample. Historically, OPV-2 has been the most-detected serotype in environmental samples [11–14]. This could be due to differences in assay, particularly for our previous work, during which our assay was more sensitive for OPV-2 than OPV-1 and -3 [13]. This could also be due to time from vaccination. As seen in our transmission studies, although OPV-2 was found in the most positive samples, OPV-3 had the longest shedding duration [15]. With the transition to bOPV in April and May 2016, new research is needed to understand the differences in the isolation of OPV serotypes from environmental samples.

We also found OPV in 2 samples before OPV administration: in February 2015 and in February 2016. The results of the February 2015 samples was confirmed via Sanger sequencing. In prior studies, we used a different PCR assay to calculate the revertant proportion (RP; the amount of OPV with well-documented mutations associated with neurovirulence) and then used the RP to determine the age of the circulating OPV [13, 19]. Unfortunately, there was too little of the virus present in these samples for the RP assay to work successfully. However, these samples are unlikely to be the result of the prior NIWs, as OPV is only administered in February and May, meaning circulation would have occurred for at least 9 months, longer than our prior study detected OPV in Mexico [13]. Additionally, for the February 2016 sample, no other environmental samples were positive from October 2015–January 2016. This implies that the OPV detected in these samples may have been imported into the community.

Our approach has a key limitation. While we were successful in detecting OPV isolates extracted from environmental samples, we did not collect river characteristics during sewage collection, such as temperature, pH, current speed, etc. As a result, we cannot add to the discussion regarding best practices when selecting locations for sewage collection. Such information will be critical in post-eradication poliovirus surveillance.

Our validation also has several strengths. First, as mentioned before, Mexico is an ideal location to study OPV, since OPV is only introduced into the community biannually. As a result, we can be confident that our OPV isolates are the result of these vaccination campaigns. Second, as these communities are primarily IPV-vaccinated, they will mimic post-eradication populations, as the global withdrawal of OPV and the transition to IPV vaccination continues.

In conclusion, our RT-PCR assay is highly sensitive and was able to detect OPV in environmental samples after only 15% of children were vaccinated across 3 different communities. Our analysis of environmental samples after unrestricted OPV vaccination additionally suggest that OPV may circulate in communities for several months in IPV-vaccinated communities. Further work should focus on environmental surveillance strategies for poliovirus, as well as how the transition from tOPV to bOPV will impact OPV isolation from environmental samples.

## Notes

***Acknowledgments.*** The authors thank the families who participated in this study and the large field teams in Mexico that performed data and sample collection, as well as the broader team at Stanford University, including Aisha Talib, Jennifer Lee Kang, and Meira Falkovitz-Halpern.

***Financial support.*** This work was supported by the Bill & Melinda Gates Foundation (grant number OPP1116910).

***Supplement sponsorship. ***This article appears as part of the supplement “Polio endgame and beyond: Vaccine choices, transmission dynamics, and surveillance implications,” sponsored by The Bill & Melinda Gates Foundation.

***Potential conflicts of interest.*** All authors: No reported conflicts of interest. All authors have submitted the ICMJE Form for Disclosure of Potential Conflicts of Interest. Conflicts that the editors consider relevant to the content of the manuscript have been disclosed.
